# Rodents: food or pests in Neolithic Orkney

**DOI:** 10.1098/rsos.160514

**Published:** 2016-10-19

**Authors:** Andrzej A. Romaniuk, Alexandra N. Shepherd, David V. Clarke, Alison J. Sheridan, Sheena Fraser, László Bartosiewicz, Jeremy S. Herman

**Affiliations:** 1 School of History, Classics and Archaeology, University of Edinburgh, Teviot Place, Edinburgh EH8 9AG, UK; 2 Skara Brae Publication Project, 509 King Street, Aberdeen AB24 3BT, UK; 3 Department of Scottish History and Archaeology, National Museums of Scotland, Chambers Street, Edinburgh EH1 1JF, UK; 4 Department of Natural Sciences, National Museums of Scotland, Chambers Street, Edinburgh EH1 1JF, UK; 5 Department of Archaeology and Classical Studies, Osteoarchaeological Research Laboratory, Stockholm University, Stockholm, Sweden

**Keywords:** Rodentia, *Microtus arvalis*, archaeology, animal osteology, human subsistence

## Abstract

Rodents have important effects on contemporary human societies, sometimes providing a source of food but more often as agricultural pests, or as vectors and reservoirs of disease. Skeletal remains of rodents are commonly found in archaeological assemblages from around the world, highlighting their potential importance to ancient human populations. However, there are few studies of the interactions between people and rodents at such sites and most of these are confined to locations where rodents have formed a part of the recent diet. Here we compare the accumulation pattern of rodent remains from four locations within and adjacent to the renowned Neolithic site of Skara Brae, Orkney, showing that those within the settlement itself were the result of deliberate human activity. The accumulation and nature of burnt bones, incorporated over an extended period within deposits of household waste, indicate that rodents were used as a nutritional resource and may have been the subject of early pest control. We, therefore, provide the first evidence for the exploitation or control of rodents by the Neolithic inhabitants of Europe.

## 1. Introduction

Rodents form the most diverse order of living mammals, with over 2000 species [1], some of which are very abundant. Between them, they have been able to exploit most terrestrial habitats and they include archetypal commensal species like the rats (*Rattus* spp.) and house mice (*Mus* spp.) [2]. Rodents may be very important to human populations, sometimes for their nutritional or even cultural value, but more often through their impact as pests of agriculture and stored food, or as reservoirs and vectors of disease [3–5].

Despite the important influence of rodents on human populations, and the presence of their skeletal remains in many archaeological assemblages, zooarchaeological evidence has rarely been used to examine the interactions between them. Some of these studies incorporate a relatively holistic view of rodent commensalism [6,7], but few have sought to examine a comprehensive range of factors that might influence the composition of rodent bone assemblages (e.g. [8,9]). The majority focus on more specific aspects, for example, the possible inclusion of rodent remains in ritual contexts [10]. Prominent among these latter studies are those which consider the use of rodents as food, particularly in the Americas [11–15], South Africa [16,17] or the Far East [18]. These are places where rodents recently formed, and sometimes still are, a part of the local diet [3]. To our knowledge, specific interactions between people and rodents, for example their utilization as a source of food, have not been studied in depth at any archaeological site within Europe. This is remarkable, given the highly developed nature of European archaeology and the potential importance of rodents to the survival of sedentary human populations.

The Orkney archipelago, situated to the north of the British mainland, is renowned for its outstanding collection of Neolithic sites, a core group of which have been recognized with World Heritage status. Among the wealth of material recovered from the Orcadian Neolithic sites are copious rodent remains from two settlements, one the celebrated site of Skara Brae, mainland [19], and the other Links of Noltland, Westray [20]. These remains are from contexts that have been radiocarbon-dated to the late fourth and early third millennium BC, using either the rodent remains themselves or other associated bone material [21,22]. Most are from the Orkney vole, a form of the European common vole *Microtus arvalis* (Pallas, 1778) that is found on some islands of the archipelago, where it is isolated from its nearest conspecifics in mainland Europe [23]. The rest are from the wood mouse *Apodemus sylvaticus* (Linnaeus, 1758), a common species found throughout western Europe and mainland Britain [24]. Neither could have survived the Pleistocene glaciations in this location and it is generally accepted that small mammals were subsequently introduced to Orkney by human agency [25]. Previous studies of the rodent remains from these sites were confined to easily identifiable skulls, mandibles and teeth, with no corresponding investigation of postcranial skeletal elements, and were concerned with the identification [26,27], origin [28,29] and evolution [30–32] of these island populations, rather than their interactions with the human population.

**Figure 1.**
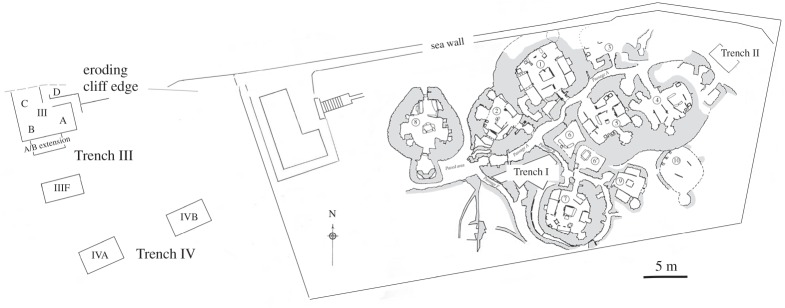
Plan of Neolithic site at Skara Brae, Orkney, showing locations of excavated trenches (I–IV on figure) and dwelling structures (shaded grey).

The excavations in 1972–1973 and 1977 at Skara Brae [19,21] yielded over 1.1 kg of sieved micromammal bone material, of which 815 g could be attributed to rodents. The excavations comprised four trenches (I–IV; figure 1), whose location and good preservation provide a remarkable opportunity to study both the human and natural deposition of remains. Trench I and its stratigraphy represents an undisturbed area inside the settlement complex, covering three phases (phase 0, 1 and 2) of human inhabitation, lasting from *ca* 3360 to *ca* 2440 cal BC, with a hiatus of some length (possibly as much as 300 years) between phase 0 and phase 1. Trench II represents the build-up of deposits at the northeastern edge of the settlement, dating from *ca* 2900 to *ca* 2490 cal BC. Unlike Trenches I and II, which are located within the core and periphery of the main settlement, respectively, Trenches III and IV were excavated 30–45 m to the west of the modern guardianship area around the settlement. These latter trenches showed only basal survival of *in situ* occupation deposits; otherwise they mainly comprised natural accumulations of sediments, principally sand with some redeposited anthropogenic material associated with interludes of plough cultivation. The dating reflects this, with dates for the basal *in situ* deposits lying between 2880 and 2470 cal BC, with later material from around 2300 to 2040 cal BC and much later activity still between cal AD 600 and 900. Given the detailed analysis of stratigraphy and the use of wet and dry sieving (meshes from 5 to 1.5 mm), it is safe to assume that most of the rodent bones were retrieved and securely attributed to their given contexts, both natural and anthropogenic.

## 2. Material and methods

The individual fragments from each context were attributed to specific bones and identified to species, where possible, using a combination of published descriptions (e.g. [33,34]) and skeletal reference material. The number of identifiable specimens (NISP) and the minimum number of elements (MNE) were recorded for each species, in each context, following a standard procedure [35,36]. Minimum number of individuals (MNI) was estimated for each species in each context, based on the MNE, excluding teeth.

Relative frequencies, observed MNE as a proportion of expected MNE, were calculated for each skeletal element in each context and trench. Expected MNE can be derived from the number of each skeletal element present in the skeleton, together with the MNI. Relative frequencies of individual elements were then averaged for each context, to give an overall measure of the relative completeness of the skeletons therein: higher values of average relative frequency will be obtained when more of the individual bones are present from each animal.

Proportions of cranial to postcranial skeletal elements, distal to proximal limb components, isolated incisor and molar teeth were obtained for each trench, following the standard procedure described in Andrews [37]. Proportions were obtained by dividing the frequency of one type of skeletal element by the other type of skeletal element, or the number of teeth by the number of empty dental alveoli, and multiplying the result by 100. Molar teeth of *M. arvalis* and *A. sylvaticus* were distinguished, but incisor teeth were not. Frequencies, obtained and expected MNE, and time span for each context and trench are shown in electronic supplementary material, table S1.

Sample size, means and standard deviations are provided for lengths of humeri and femora. Age pyramids [38] show these elements categorized by length in 1 mm intervals and were determined only for the dominant assemblages in Trenches I and IV, where considerable numbers of remains were deposited within a short period of time, presumably by the same agent. All measurements are summarized in electronic supplementary material, table S2.

Fragmentation of skeletal elements was determined by standard procedure [37] and the overall breakage pattern was established for each trench. The observed fragmentation, mostly around the D or C class of breakage for maxillary/skull elements, could have occurred at or before deposition or could be due to subsequent soil movement inside the Skara Brae settlement [39].

Methods using weights of assemblages and individual bones [35,36] were not applied here, due to the level of fragmentation and the difficulty in accurately weighing so many small fragments. Similarly, sex could be determined in less than 4% of the remains and only the presence of both sexes was noted.

For quantitative data, *χ*^2^-statistics were used to examine the differences between observed and expected, or target, values. Observed values of both NISP and MNI were compared with the values expected under a null hypothesis, that these were proportionately distributed among Trenches I–IV (table 1). For each trench at Skara Brae, both skeletal element relative frequencies and long bone fragmentation were compared with equivalent values in the remaining trenches, or published values [13,17,37] attributed to a range of depositional agents (see electronic supplementary material, table S3).

**Table 1:** Number of identified specimens (NISP) and minimum number of individuals (MNI) from each trench. *χ*^2^-tests (NISP; *χ*^2^ = 54 593, d.f. 3, *p* < 0.001) and MNI (MNI; *χ*^2^ = 3512.95, d.f. 3, *p* < 0.001) reject null hypothesis of equal distribution among trenches.

Trench	region of settlement	volume (m^3^)	NISP	NISP m^−3^	MNI	MNI m^−3^
I	core	53.15	22 359	420.67	1340	25.21
II	periphery	23.32	1032	44.25	74	3.17
III	off-site	124.85	110	0.88	25	0.20
IV	off-site	45.00	7023	156.07	235	5.22
total	—	246.32	30 524	—	1674	—

*χ*^2^-tests may not be reliable when applied to NISP or MNI, due to the inherent properties of such data. However, of the two measures, MNI will be less affected by differential survivability and fragmentation of skeletal elements and may, therefore, be preferable. Furthermore, given that the data for each trench comprised data from numerous contexts, the results of all these tests should be treated with caution, particularly with regard to measures of significance. Nevertheless, they may at least provide some insight into the relative divergence of the samples here from alternative patterns: lower values of the *χ*^2^ statistic will reflect greater similarity.

## 3. Results and discussion

### 3.1. Distribution and composition of rodent skeletal remains

The sieved microskeletal material included 816 g of identifiable rodent remains. These comprised 30 524 skeletal fragments, including 8369 isolated teeth, attributable to at least 1674 individual animals. Only two species were identified, Orkney vole and wood mouse, supporting the notion that only these two rodents had been introduced to Orkney by the time of the Neolithic [26,27].

Quantitative comparisons of the rodent bones between trenches and their constituent contexts highlight differences, most notably the marked accumulation of remains within the settlement itself. Most of the remains (73% NISP) and individual animals (80% MNI) were extracted from largely anthropogenic contexts within the settlement itself (table 1). The bias towards the anthropogenic contexts of Trench I is still apparent, after accounting for the volume of material extracted from each trench (table 1). Observed values for NISP and MNI, taking account of context size, are significantly different (*p* < 0.001) from expected values (table 1), although this conclusion should be treated with caution (see Material and methods).

**Figure 2.**
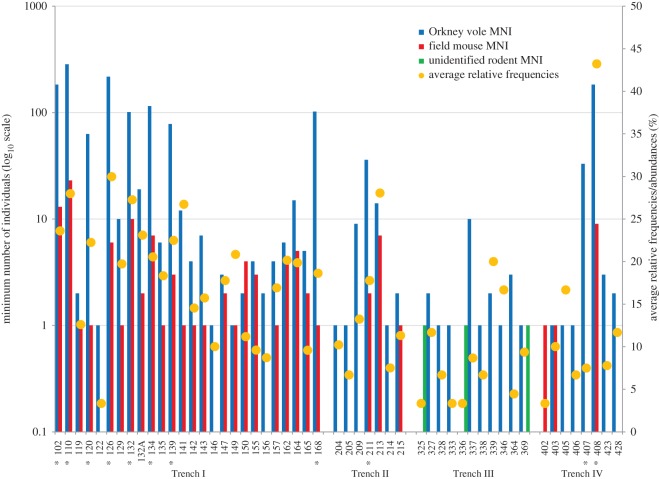
Average relative frequency/abundance (a measure of skeletal completeness) and minimum number of individuals (MNI) for each species on log_10_ scale (* indicates contexts with MNI greater than mean).

Moreover, additional differences are highlighted when single contexts are analysed. Among the 52 assemblages examined, only 11 contexts had MNI values above the overall mean (greater than 32), and eight of these contexts were located within the main site (figure 2). Differences in values between Trench I and the rest of the studied area are also visible in the case of smaller contexts, which usually contain far more than one or two specimens. The bias in accumulation is even more marked than it would appear from these MNI values, given that Trench I includes a large proportion of contexts of comparatively small volume.

Average relative frequencies (abundances) of skeletal elements for each context also show a higher skeletal completeness in the centre of the site (figure 2). While this pattern is expected for larger skeletal samples [35,36], skeletons from smaller assemblages in Trench I are also more complete than those from peripheral (Trench II) or off-site (Trenches III and IV) contexts of similar size. Interestingly, one context from Trench IV (number 408) has a very high average relative frequency, differing not only from other contexts within that trench but also from those in Trench I.

There are also differences in the numbers and distribution of wood mice and Orkney voles in each trench, and among contexts within them (figure 2). Voles are almost ubiquitous, being found in all but two of the 52 contexts, but with MNI varying from one to several hundreds. By contrast, the wood mouse population seems to be more biased towards the settlement than that of the voles, because no mouse bones were found in the off-site Trench III and very few were found in the two largest contexts of the off-site Trench IV. However, in contexts where it does occur, the numbers of wood mouse are more consistent, with MNI generally less than five individuals.

**Figure 3.**
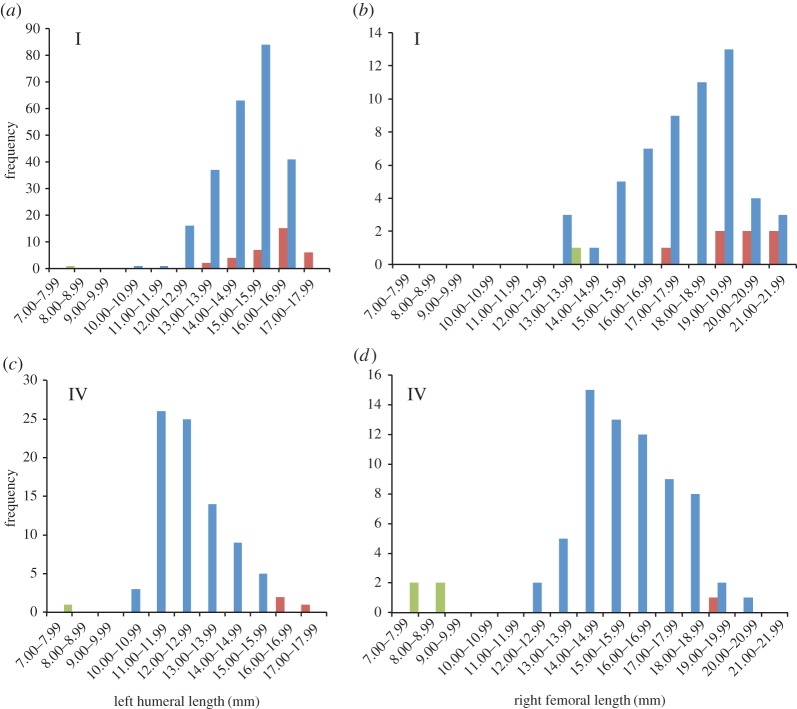
(*a*--*d*) Lengths of Orkney vole humeri and femora from Trenches I and IV. Both epiphyses fused (red), proximal epiphysis unfused (blue) and both epiphyses unfused (green).

Age pyramids for voles from the two largest assemblages, Trench I in the core settlement and off-site Trench IV, show similarities and also some differences (figure 3). Few juvenile bones were recovered from either trench, with Trench I producing only one measurable juvenile femur and no humeri with both epiphyses unfused, while Trench IV contained only five juvenile bones. The proportion of fully fused bones, from mature adults, was higher in Trench I than in Trench IV. Perhaps more importantly, given the relative numbers of adult and immature animals preserved, Trench I also contained more bones that approached adult size, among the partially fused bones that had come from immature animals. Whereas the pattern in Trench IV might well reflect a ‘natural’ distribution from a freely roaming population, that of Trench I is biased towards older animals and unlikely to do so. Although this does not have any influence on the patterns described above, it is notable that most of the long bone finds from Trench IV were measurable, whereas only a small proportion (approx. 15%) of those from Trench I were complete enough for this purpose. Once again, this highlights the difference in depositional pattern, and therefore the likely agent, between these contexts from within and outside of the settlement.

These differences in numbers, skeletal completeness and age profiles of common vole bones recovered from the settlement, its periphery and the off-site areas, together show that the pattern of deposition was localized, rather than random. It is, therefore, unlikely that the remains are from accidently trapped microvertebrates, which are frequently encountered on archaeological sites [40,41]. It can also be deduced that the majority of Orkney vole remains in Trenches I and II were deposited during context formation, as no intrusive burrowing marks were identified, no dense deposits of rodent remains recovered and the individual rodent elements were commingled with other context deposits. In addition, anthropogenic sediments from Trench I revealed a lack of organic content [39], as would be expected if there was substantial animal activity or a large number of decaying bodies present. Given that common voles inhabit open areas, especially rough grasslands [23], it is highly unlikely that they were resident inside an occupied human settlement which relied mainly on marine and terrestrial animals as a food source [42]. Wood mice, by contrast, seasonally enter human dwellings [24] and their presence in so many contexts from the core settlement may reflect this.

### 3.2. Agent and nature of skeletal deposition

The only explanation for the consistent presence of vole bones in Trenches I and II, often in large numbers, is that predatory animals or humans were responsible for their deposition. This is particularly so for those contexts with large numbers of bones that are relatively complete, as these features indicate primary deposition by such taphonomic agents [35,36]. However, the bias towards these trenches also reveals that deposition is unlikely to be the result of animal predation alone, because these large accumulations are generally confined to the core site, rather than dispersed around and within it, as might be expected if they followed the distribution of predators within the landscape [14]. Only context 408 from Trench IV, with a high relative frequency of bone elements and a more natural age pyramid, could be due to a wild predator, but this pattern of deposition might equally be due to other factors, for example, intrusive burrowing, episodes of which were closely associated with this context.

**Figure 4.**
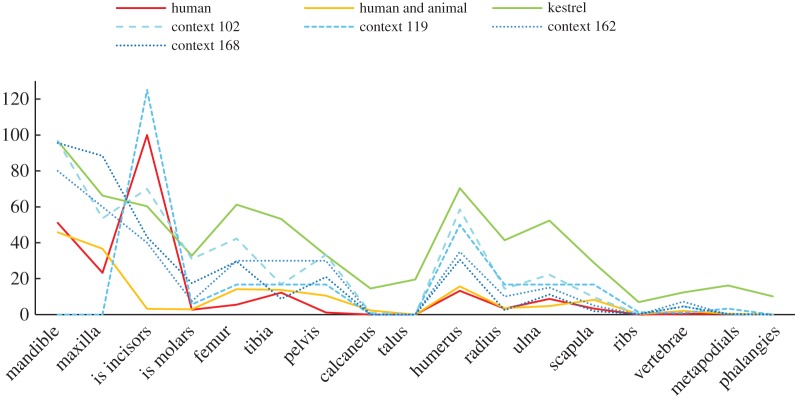
Relative frequencies (abundances) for four selected contexts from Trench I and three patterns similar to them: human processing [17], human and animal [13], kestrel [37]. Both species combined.

**Figure 5.**
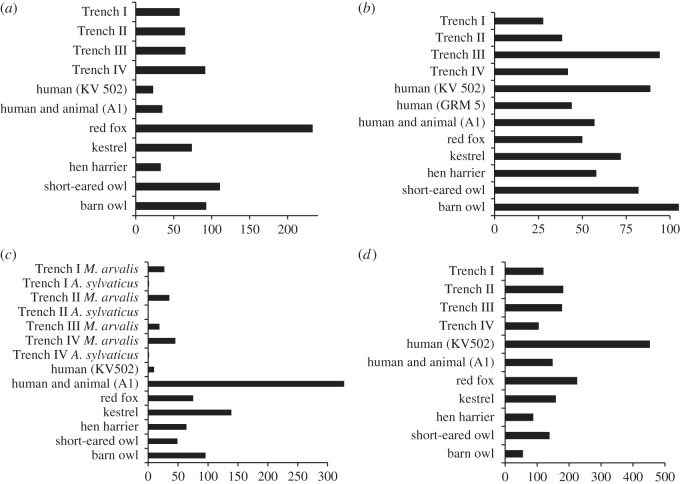
Proportions (expressed as %) of different skeletal elements and of isolated teeth in each trench and in sites elsewhere. Animal patterns after Andrews [37], human (KV 502, GRM 5) after Dewar & Jerardino [17], human and animal (A1) after Fernandez *et al.* [13]. (*a*) Proportion of postcranial to cranial elements; both species combined. (*b*) Proportion of distal to proximal limb elements; both species combined. (*c*) Proportion of isolated molar teeth. (*d*) Proportion of isolated incisor teeth; both species combined.

Determination of the actual agents of deposition is not straightforward in the case of these Skara Brae remains. Standard quantitative methods of analysis are handicapped by the lack of comparative data from Orkney and the number of contexts which should be considered independently. With regard to the overall frequency of each skeletal element (figure 4), the depositional pattern in Trench I seems to be more similar to that of the kestrel (*Falco tinnunculus* Linnaeus, 1758) than to those from other predatory species that inhabit Orkney [37,43], especially in the case of the larger assemblages (contexts 102, 162, 168). However, frequencies of some skeletal elements and the overall proportions are closer to that from human combined with animal deposition [13]. In smaller contexts, for example, context 119, they may even show similarities with human processing [17]. The proportions of specific skeletal parts, for example, cranial to postcranial or proximal to distal extremities of bones, as well as the proportions of isolated incisors or molars [37], also resemble those from the kestrel [37] or human and animal [13] deposition (figure 5). Fragmentation of skeletal elements (electronic supplementary material, table S4) was intermediate between the relatively high level of breakage produced by mammalian carnivores, such as canids, and the limited breakage that results from owl predation [37,43]. Digestion marks were present but rare, especially on teeth or cranial elements, and were similar to those produced by owls [37]. However, this pattern could also be the result of human processing or the dismemberment of rodents prior to their consumption by animals [43]. In any case, evidence from fragmentation and digestion should be treated with caution here, given the possible effects of soil perturbation at the Skara Brae site [39].

Burn marks were present on some of the rodent bones from Trenches I and II, providing further evidence of human involvement in their deposition. Approximately 311 charred bone and tooth fragments were retrieved from 14 out of 26 contexts in Trench I, in frequencies relative to the size of the assemblage, implying the repeated incineration of rodent bones throughout the period of occupation. Although bones may be affected by heat from natural fires [35,44], this is highly unlikely to be the case here, given the absence of burnt remains in the off-site Trenches III and IV and the presence of only four burnt elements in the peripheral Trench II, together with the distribution of burning over several centuries and the nature of the Orcadian climate, which is not conducive to spontaneous fires. Within the settlement, the spatial correlation of burnt elements with anthropogenic contexts provides further evidence that humans were responsible for their modification [13,17,45].

**Figure 6.**
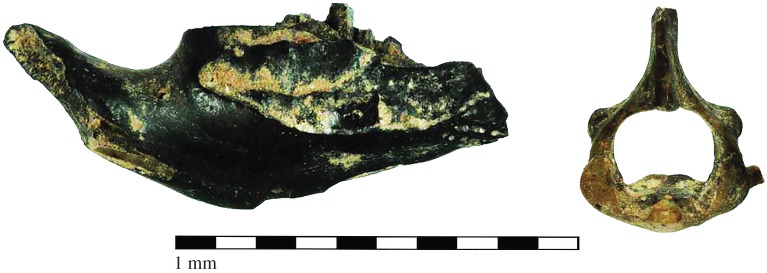
Charred vole mandible and partially burnt atlas vertebra from Trench I.

Several characteristics of the burn marks also indicate that incineration of the voles was related to intentional handling. The superficial appearance of charred bones will depend on a number of factors, including the length of the process and the temperature [46]. The Skara Brae remains appear to have been charred rapidly at a high temperature (figure 6). Although bone calcination may be the outcome of intentional human action [47], it can be due to thermal alteration of established deposits [44]. However, the latter process would produce a large proportion of calcined bone fragments, including those from other species present in the assemblage, whereas most of the burnt bones and teeth from Skara Brae were only carbonized and all but one, a wood mouse femur, were attributable to Orkney voles. Moreover, burn marks are usually distributed unevenly over the surface of bones from established deposits, due to the uneven distribution of heat within the soil, while directly incinerated specimens usually exhibit evenly discoloured surfaces [46]. Burn marks on the vole remains from Skara Brae are confined to the more exposed surfaces of the bones and teeth, especially in the case of mandibles, molar and incisor teeth. The discoloration is evenly spread as a dark stain over most of the surface, implying that the temperature was stable and evenly distributed, which is unlikely if they were incinerated after deposition in the soil [46]. However, there are a number of partially burnt bones, mostly humeri, femora and cervical vertebrae. Charring marks are unevenly distributed across these bones, including the articular surfaces of the joints. The overall pattern, combining the even charring of exposed surfaces with the uneven discolouration of internal elements, may indicate that the animals were burnt while still in a partially articulated state, with some of the soft tissue intact [35,36], as previously suggested for other sites where human or mixed human–animal deposition were implicated [11,13,16,17]. In addition, the burnt and unburnt remains were commingled, inside as well as outside the hearth context, which is highly unlikely if the burn marks were the result of incineration after their deposition in the soil.

The evidence from Skara Brae indicates that the accumulation and burning of Orkney vole remains were most likely the result of deliberate actions by the inhabitants of the settlement, carried out over a considerable period of time, and the most plausible explanation is the utilization of the voles as a source of food. It is notable that the rodent remains were not found in isolation, but within deposits that contained waste products from a range of other foods, including mammals, fish and shellfish. The presence of bones within the hearth deposit itself may be due to roasting of voles between the embers, as previously seen in two other regions of the world, South Africa and Patagonia [13,16]. Another possibility is cooking or boiling of animals in a pot, known from some ethnographic studies [48] and also suggested for assemblages from the Pampean region in Argentina [15]. In all of these cases, the burnt elements include mandibles and teeth [15,16,18,49], as in the case of the Skara Brae material. The predominance of older individuals in the sample from Skara Brae may point towards selective or seasonal ‘hunting’, which would not require any more sophisticated techniques than pit-trapping. The proportion of such finds at Skara Brae (1% of fragments in Trench I), is lower than that from other studied contexts [11,14,16,17], but their number may depend on the specific preparation methods that were used in each location [15,48].

It is also possible that the vole remains are the outcome of agricultural pest control, although it is unlikely that this was their only source, given their location and intermingling with household refuse. However, rodent consumption and control are not mutually exclusive and it is plausible that captured pest animals were being eaten, a frequent practice today [3]. Surprisingly, previous studies of early pest control have concentrated on invertebrates [50,51], despite the serious impact of rodents on stored food and on human health.

## 4. Conclusion

This is the first indication that any species of rodent was exploited by the Neolithic inhabitants of Europe, although other species of rodent have been exploited there in more recent times, for example, the edible dormouse (*Glis glis* Linnaeus, 1766) which was a popular delicacy, raised in *gliaria* in the Roman Empire [3,52]. Orkney voles were introduced from continental Europe during the Neolithic, although the precise source of the animals has been disputed [22,28,29]. The evidence for their exploitation presented here might offer some indirect support to the idea that the voles were knowingly transported from the Continent, quite possibly as a food resource during long sea journeys [28,29,31], especially when sufficient numbers were introduced to enable the establishment of genetically diverse populations [28,31]. Perhaps this might also explain, through the idea of funerary rites featuring the consumption and offering of food, why vole remains were found in a chambered cairn on the Holm of Papa Westray [53], as well as in other sites on smaller islands where common voles do not presently exist.

## Supplementary Material

Table S1. Skeletal elements and radiocarbon dates for each trench and context.

## Supplementary Material

Table S2. Measurements of skeletal elements. Table S3. Relative frequencies of skeletal elements. Table S4. Fragmentation of remains

## Data accessibility

The data supporting this article are available from the Dryad Digital Repository at http://dx.doi.org/10.5061/dryad.j333r [54], and in the electronic supplementary information.
