# Supplementary material for: Rodents: food or pests in Neolithic Orkney
Source: R Soc Open Sci. 2016 Oct 19;3(10):160514. doi: 10.1098/rsos.160514 (PMC5098993; doi:10.1098/rsos.160514)
Supplement: Table S2. Measurements of skeletal elements. Table S3. Relative frequencies of skeletal elements. Table S4. Fragmentation of remains [file rsos160514supp2.pdf]

Table S2. Measurements of Orkney vole skeletal elements (mm) from Trench I (contexts 102, 110, 120, 126, 132, 134, 139 and 168) and Trench IV (context 408).

Left humerus

	Mean	SD	Max	Min	n
Trench I - Proximal epiphyses unfused	14.9	1.2	16.9	10.4	243
Trench I - Both epiphyses fused	16.0	1.1	17.7	13.4	34
Trench IV - Proximal epiphyses unfused	13.7	1.3	16.9	11.5	82
Trench IV - Both epiphyses fused		17.4; 17.9; 18.2			3
Trench IV - Both epiphyses unfused		8.5			1

Right femur

	Mean	SD	Max	Min	n
Trench I -distal epiphysis unfused	18.1	1.8	21.8	13.9	56
Trench I - both epiphyses fused	20.2	1.3	21.7	17.3	7
Trench I - both epiphyses unfused			13.2		
Trench IV - distal epiphysis unfused	16.1	1.8	20.5	12.9	67
Trench IV - both epiphyses fused			19.6		
Trench IV - both epiphyses unfused	7.7	0.7	8.6	7.0	4

Right lower tooth row length

	Mean	SD	Max	Min	n
Trench I	7.17	0.37	7.98	6.3	55
modern sample	6.22	0.52	6.63	5.14	10

Table S3. Comparison of skeletal element relative frequencies (SERF; %, $\chi2$, df 16) and long bone fragmentation (FRAG; %, $\chi2$, df 15); both species combined.

	Trench I		Trench II		Trench III		Trench IV	
	SERF	FRAG	SERF	FRAG	SERF	FRAG	SERF	FRAG
Trench II	8.65	20.70						
Trench III	44.80	182.26	30.36	150.66				
Trench IV	32.75	33.58	28.67	26.70	48.78	193.41		
barn owl (37)	276.33	529.81	236.87	489.48	128.78	300.50	224.91	474.82
short-eared owl (37)	177.41	431.97	138.88	393.2	67.54	272.60	151.68	381.41
kestrel (37)	98.70	51.42	77.54	59.05	42.15	185.06	94.08	47.58
hen harrier (37)	83.70	121.91	80.35	111.36	51.61	254.56	150.21	111.26
red fox (37)	186.46	145.37	142.66	210.47	80.25	446.89	166.66	189.85
human and animal (13)	37.31	155.39	41.76	195.72	39.83	414.24	42.74	209.52
human processing (17)	125.05		122.55		84.52		207.98	
human digestive (17)	494.49		434.60		334.71		405.26	

Table S4. Fragmentation in contexts from each trench; both species combined.

Trench I

Breakage of skull	n	%
Skulls (all broken)	38	1.1
Maxillary with zygomatic	458	12.9
Maxillary without zygomatic	1574	44.4
Minor fragments	1475	41.6
Molars missing from maxillary	6056	97.6
Incisors missing from premaxillary	684	94.7

Breakage of mandible	n	%
Complete mandible	74	3.0
Ascendant ramus broken	363	14.9
Without ascendant ramus	789	32.3
Without ascendant ramus and inferior edge broken	1218	49.8
Molars missing from mandible	6552	89.0
Incisors missing from mandible	1852	75.9

Breakage of postcranial elements	n	%
Humerus - complete	727	41.1
Humerus - proximal	226	12.8
Humerus - shaft	230	13.0
Humerus - distal	585	33.1
Ulna - complete	185	21.3
Ulna - proximal	374	43.0
Ulna - shaft	211	24.3
Ulna - distal	99	11.4
Femur - complete	283	13.2
Femur - proximal	1012	47.3
Femur - shaft	602	28.1
Femur - distal	244	11.4
Tibia - complete	83	9.2
Tibia - proximal	415	46.0
Tibia - shaft	301	33.4
Tibia - distal	103	11.4

Trench II

Breakage of skull	n	%
Skulls (all broken)	0	0.0
Maxillary with zygomatic	10	9.6
Maxillary without zygomatic	57	54.8
Minor fragments	37	35.6
Molars missing from maxillary	201	100.0
Incisors missing from premaxillary	2	100.0

Breakage of mandible	n	%
Complete mandible	0	0.0
Ascendant ramus broken	23	19.0
Without ascendant ramus	48	39.7
Without ascendant ramus and inferior edge broken	50	41.3
Molars missing from mandible	321	88.4
Incisors missing from mandible	70	57.9

Breakage of postcranial elements	n	%
Humerus - complete	35	38.0
Humerus - proximal	17	18.5
Humerus - shaft	17	18.5
Humerus - distal	23	25.0
Ulna - complete	8	27.6
Ulna - proximal	9	31.0
Ulna - shaft	9	31.0
Ulna - distal	3	10.4
Femur - complete	22	22.0
Femur - proximal	40	40.0
Femur - shaft	28	28.0
Femur - distal	10	10.0
Tibia - complete	7	10.6
Tibia - proximal	21	31.8
Tibia - shaft	20	30.3
Tibia - distal	18	27.3

Trench III

Breakage of skull	n	%
Skulls (all broken)	0	0.0
Maxillary with zygomatic	2	25.0
Maxillary without zygomatic	6	75.0
Minor fragments	0	0.0
Molars missing from maxillary	20	83.3
Incisors missing from premaxillary	0	X

Breakage of mandible	n	%
Complete mandible	0	0.0
Ascendant ramus broken	3	15.8
Without ascendant ramus	6	31.6
Without ascendant ramus and inferior edge broken	10	52.6
Molars missing from mandible	39	68.4
Incisors missing from mandible	9	47.4

Breakage of postcranial elements	n	%
Humerus - complete	7	100.0
Humerus - proximal	0	0.0
Humerus - shaft	0	0.0
Humerus - distal	0	0.0
Ulna - complete	2	50.0
Ulna - proximal	0	0.0
Ulna - shaft	1	25.0
Ulna - distal	1	25.0
Femur - complete	5	33.3
Femur - proximal	3	20.0
Femur - shaft	4	26.7
Femur - distal	3	20.0
Tibia - complete	3	21.4
Tibia - proximal	4	28.6
Tibia - shaft	4	28.6
Tibia - distal	3	21.4

Trench IV

Breakage of skull	n	%
Skulls (all broken)	18	2.3
Maxillary with zygomatic	88	11.5
Maxillary without zygomatic	273	35.7
Minor fragments	386	50.5
Molars missing from maxillary	1115	98.1
Incisors missing from premaxillary	184	89.3

Breakage of mandible	n	%
Complete mandible	21	4.7
Ascendant ramus broken	150	33.9
Without ascendant ramus	172	38.8
Without ascendant ramus and inferior edge broken	100	22.6
Molars missing from mandible	1234	92.9
Incisors missing from mandible	317	72.2

Breakage of postcranial elements	n	%
Humerus - complete	196	29.9
Humerus - proximal	77	11.8
Humerus - shaft	201	30.7
Humerus - distal	181	27.6
Ulna - complete	48	18.2
Ulna - proximal	119	45.3
Ulna - shaft	81	30.8
Ulna - distal	15	5.7
Femur - complete	180	31.4
Femur - proximal	186	32.5
Femur - shaft	159	27.7
Femur - distal	48	8.4
Tibia - complete	95	23.7
Tibia - proximal	135	33.7
Tibia - shaft	129	32.2
Tibia - distal	42	10.4
