# Supplementary material for: Rodents: food or pests in Neolithic Orkney
Source: R Soc Open Sci. 2016 Oct 19;3(10):160514. doi: 10.1098/rsos.160514 (PMC5098993; doi:10.1098/rsos.160514)
Supplement: Table S1. Skeletal elements and radiocarbon dates for each trench and context. [file rsos160514supp1.pdf]

Table S.1. For each trench and context: obtained minimum number of elements (MNE), minimum number of individuals (MNI) per species, expected MNE based on MNI, relative frequencies (abundance) based on obtained and expected MNE, mean frequencies and radiocarbon dating (95.4% probability)11.

Overall	Trench I		
	Obtained MNE	Expected MNE	Relative Abundance
Maxilla	2070	2680	77.2%
Mandible	2463	2680	91.9%
Scapula	294	2680	36.4%
Pelvis	976	2680	36.4%
Humerus	1331	2680	49.7%
Ulna	551	2680	20.6%
Radius	220	2680	8.2%
Femur	1294	2680	48.3%
Tibia	509	2680	19.0%
Vertebrae	3332	49580	6.7%
Calcaneus	5	2680	0.2%
Talus	2	2680	0.1%
Ribs	53	2680	0.2%
Metapodials	128	26800	0.5%
Phalangies	5	300160	0.0%
Isolated Incisors	3164	5360	59.0%
Isolated Molars	3303	16080	20.5%
Minimum Number of Individuals (M.arvalis/A.sylvaticus/Unidentified)	1246/94/-		
Calibrated Dating	3350-2290 B.C.		

Trench I contexts	Context 102			Context 110			Context 119			Context 120			Context 122		
	Obtained MNE	Expected MNE	Relative Abundance	Obtained MNE	Expected MNE	Relative Abundance	Obtained MNE	Expected MNE	Relative Abundance	Obtained MNE	Expected MNE	Relative Abundance	Obtained MNE	Expected MNE	Relative Abundance
Maxilla	209	390	53.6%	593	614	95.6%	0	6	0.0%	98	128	76.6%	0	2	0.0%
Mandible	377	390	96.7%	564	614	91.9%	0	6	0.0%	121	128	94.5%	0	2	0.0%
Scapula	38	390	9.7%	76	614	12.4%	1	6	16.7%	15	128	11.7%	0	2	0.0%
Pelvis	130	390	33.3%	248	614	40.4%	1	6	16.7%	47	128	36.7%	0	2	0.0%
Humerus	228	390	58.5%	362	614	59.0%	3	6	50.0%	50	128	39.1%	0	2	0.0%
Ulna	87	390	22.3%	143	614	23.3%	3	6	50.0%	18	128	14.1%	0	2	0.0%
Radius	56	390	14.4%	53	614	8.6%	1	6	16.7%	5	128	3.9%	0	2	0.0%
Femur	165	390	42.3%	365	614	59.5%	1	6	16.7%	42	128	32.8%	0	2	0.0%
Tibia	66	390	16.9%	123	614	20.0%	1	6	16.7%	18	128	14.1%	1	2	50.0%
Vertebra	287	6240	4.6%	821	9824	8.4%	1	96	1.0%	193	2048	9.4%	0	32	0.0%
Calcaneus	4	390	1.0%	0	614	0.0%	0	6	0.0%	1	128	0.8%	0	2	0.0%
Talus	0	390	0.0%	1	614	0.2%	0	6	0.0%	0	128	0.0%	0	2	0.0%
Ribs	11	5070	0.2%	7	7982	0.1%	1	78	1.3%	1	1664	0.1%	0	26	0.0%
Metapodials	17	3900	0.4%	21	6140	0.3%	2	60	3.3%	0	1280	0.0%	0	20	0.0%
Phalanges	0	10920	0.0%	1	17192	0.01%	0	168	0.0%	0	3584	0.0%	0	56	0.0%
Isolated Incisors	546	780	70.0%	925	1228	75.3%	15	12	125.0%	105	256	41.0%	0	4	0.0%
Isolated Molars	726	2340	31.0%	887	3684	24.1%	2	36	5.6%	93	768	12.1%	0	12	0.0%
Average relative frequency (excluding isolated teeth)	23.6%			28.0%			12.6%			22.3%			3.3%		
Average relative frequency (all)	26.8%			30.5%			18.8%			22.8%			2.9%		
Minimum Number of Individuals (M.arvalis/A.sylvaticus/Unidentified)	183/13/-			284/23/-			2/1/-			63/1/-			1/-/-		
Calibrated Dating (95,4%)	2840-2290 B.C.			2840-2460 B.C.			2880-2580 B.C.			------------------			2880-2580 B.C.		

Trench I contexts	Context 126			Context 129			Context 132			Context 132A			Context 134		
	Obtained MNE	Expected MNE	Relative Abundance	Obtained MNE	Expected MNE	Relative Abundance	Obtained MNE	Expected MNE	Relative Abundance	Obtained MNE	Expected MNE	Relative Abundance	Obtained MNE	Expected MNE	Relative Abundance
Maxilla	435	446	97.5%	15	22	68.2%	168	222	75.7%	22	42	52.4%	175	244	71.7%
Mandible	435	446	97.5%	15	22	68.2%	205	222	92.3%	41	42	97.6%	235	244	96.3%
Scapula	93	446	20.9%	1	22	4.5%	14	222	6.3%	5	42	11.9%	14	244	5.7%
Pelvis	235	446	52.7%	3	22	13.6%	88	222	39.6%	10	42	23.8%	77	244	31.6%
Humerus	230	446	51.6%	8	22	36.4%	129	222	58.1%	18	42	42.9%	69	244	28.3%
Ulna	119	446	26.7%	2	22	9.1%	65	222	29.3%	5	42	11.9%	24	244	9.8%
Radius	44	446	9.9%	1	22	4.6%	24	222	10.8%	5	42	11.9%	5	244	2.1%
Femur	246	446	55.2%	9	22	40.9%	135	222	60.8%	25	42	59.5%	107	244	43.9%
Tibia	108	446	24.2%	8	22	36.4%	58	222	26.1%	11	42	26.2%	28	244	11.5%
Vertebra	921	7136	12.9%	32	352	9.1%	332	3552	9.4%	45	672	6.7%	282	3904	7.2%
Calcaneus	0	446	0.0%	1	22	4.5%	0	222	0.0%	0	42	0.0%	0	244	0.0%
Talus	0	446	0.0%	0	22	0.0%	0	222	0.0%	0	42	0.0%	0	244	0.0%
Ribs	7	5798	0.1%	1	286	0.4%	0	2886	0.0%	0	546	0.0%	0	3172	0.0%
Metapodials	21	4460	0.5%	0	220	0.0%	8	2220	0.4%	8	420	1.9%	1	2440	0.04%
Phalanges	0	12488	0.0%	0	616	0.0%	0	6216	0.0%	0	1176	0.0%	0	6832	0.0%
Isolated Incisors	513	892	57.5%	6	44	13.6%	291	444	65.5%	57	84	67.9%	193	488	39.6%
Isolated Molars	580	2676	21.7%	7	132	5.3%	254	1332	19.1%	48	252	19.1%	212	1464	14.5%
Average relative frequency (excluding isolated teeth)	30.0%			19.7%			27.3%			23.1%			20.5%		
Average relative frequency (all)	31.1%			18.5%			29.0%			25.5%			21.3%		
Minimum Number of Individuals (M.arvalis/A.sylvaticus/Unidentified)	217/6/-			10/1/-			101/10/-			19/2/-			115/7/-		
Calibrated Dating (95,4%)	2890-2500 B.C.			2880-2620 B.C.			2880-2480 B.C.			-------------------			-------------------		

Trench I contexts	Context 135			Context 139			Context 141			Context 142			Context 143		
	Obtained MNE	Expected MNE	Relative Abundance	Obtained MNE	Expected MNE	Relative Abundance	Obtained MNE	Expected MNE	Relative Abundance	Obtained MNE	Expected MNE	Relative Abundance	Obtained MNE	Expected MNE	Relative Abundance
Maxilla	11	14	78.6%	88	162	54.30%	22	26	84.6%	0	10	0.0%	6	16	37.5%
Mandible	6	14	42.9%	151	162	93.20%	21	26	80.8%	7	10	70.0%	12	16	75.0%
Scapula	1	14	7.1%	20	162	12.40%	6	26	23.1%	0	10	0.0%	0	16	0.0%
Pelvis	1	14	7.1%	42	162	25.90%	13	26	50.0%	3	10	30.0%	0	16	0.0%
Humerus	9	14	64.3%	89	162	54.90%	18	26	69.2%	5	10	50.0%	4	16	25.0%
Ulna	3	14	21.4%	38	162	23.50%	0	26	0.0%	1	10	10.0%	0	16	0.0%
Radius	1	14	7.1%	9	162	5.60%	1	26	3.9%	0	10	0.0%	0	16	0.0%
Femur	5	14	35.7%	77	162	47.50%	13	26	50.0%	2	10	20.0%	5	16	31.3%
Tibia	1	14	7.1%	27	162	16.70%	6	26	23.1%	3	10	30.0%	8	16	50.0%
Vertebra	8	224	3.6%	80	2592	3.1%	67	416	16.1%	11	160	6.9%	2	256	0.8%
Calcaneus	0	14	0.0%	0	162	0.0%	0	26	0.0%	0	10	0.0%	0	16	0.0%
Talus	0	14	0.0%	0	162	0.0%	0	26	0.0%	0	10	0.0%	0	16	0.0%
Ribs	0	182	0.0%	1	2106	0.1%	0	338	0.0%	0	130	0.0%	0	208	0.0%
Metapodials	0	140	0.0%	5	1620	0.3%	0	260	0.0%	1	100	1.0%	2	160	1.3%
Phalanges	0	392	0.0%	0	4536	0.0%	0	728	0.0%	0	280	0.0%	0	448	0.0%
Isolated Incisors	13	28	46.4%	200	324	61.7%	16	52	30.8%	0	20	0.0%	14	32	43.8%
Isolated Molars	0	84	0.0%	160	972	16.5%	21	156	13.5%	1	60	1.7%	10	96	10.4%
Average relative frequency (excluding isolated teeth)	18.3%			22.5%			26.7%			14.5%			14.7%		
Average relative frequency (all)	18.9%			24.4%			26.1%			12.9%			16.2%		
Minimum Number of Individuals (M.arvalis/A.sylvaticus/Unidentified)	6/1/-			78/3/-			12/1/-			4/1/-			7/1/-		
Calibrated Dating (95,4%)	2910-2620 B.C.			2880-2480 B.C.			2870-2500 B.C.			3020-2630 B.C.			-------------------		

Trench I contexts	Context 146			Context 147			Context 149			Context 150			Context 155		
	Obtained MNE	Expected MNE	Relative Abundance	Obtained MNE	Expected MNE	Relative Abundance	Obtained MNE	Expected MNE	Relative Abundance	Obtained MNE	Expected MNE	Relative Abundance	Obtained MNE	Expected MNE	Relative Abundance
Maxilla	0	2	0.0%	0	10	0.0%	2	4	50.0%	0	12	0.0%	0	14	0.0%
Mandible	1	2	50.0%	3	10	30.0%	3	4	75.0%	8	12	66.7%	10	14	71.4%
Scapula	0	2	0.0%	2	10	20.0%	0	4	0.0%	0	12	0.0%	2	14	14.3%
Pelvis	0	2	0.0%	4	10	40.0%	2	4	50.0%	4	12	33.3%	3	14	21.4%
Humerus	1	2	50.0%	4	10	40.0%	1	4	25.0%	2	12	16.7%	3	14	21.4%
Ulna	0	2	0.0%	4	10	40.0%	0	4	0.0%	3	12	25.0%	0	14	0.0%
Radius	0	2	0.0%	2	10	20.0%	1	4	25.0%	1	12	8.3%	0	14	0.0%
Femur	0	2	0.0%	1	10	10.0%	3	4	75.0%	0	12	0.0%	0	14	0.0%
Tibia	1	2	50.0%	5	10	50.0%	0	4	0.0%	2	12	16.7%	2	14	14.3%
Vertebra	0	32	0.0%	9	160	5.6%	8	64	12.5%	0	192	0.0%	3	224	1.3%
Calcaneus	0	2	0.0%	0	10	0.0%	0	4	0.0%	0	12	0.0%	0	14	0.0%
Talus	0	2	0.0%	0	10	0.0%	0	4	0.0%	0	12	0.0%	0	14	0.0%
Ribs	0	26	0.0%	0	130	0.0%	0	52	0.0%	0	156	0.0%	0	182	0.0%
Metapodials	0	20	0.0%	10	100	10.0%	0	40	0.0%	1	120	0.8%	0	140	0.0%
Phalanges	0	56	0.0%	2	280	0.7%	0	112	0.0%	0	336	0.0%	0	392	0.0%
Isolated Incisors	2	4	50.0%	4	20	20.0%	1	8	12.5%	6	24	25.0%	5	28	17.9%
Isolated Molars	0	12	0.0%	2	60	3.3%	0	24	0.0%	1	72	1.4%	3	84	3.6%
Average relative frequency (excluding isolated teeth)	10.0%			17.8%			20.8%			11.2%			9.6%		
Average relative frequency (all)	11.8%			17.0%			19.1%			11.4%			9.7%		
Minimum Number of Individuals (M.arvalis/A.sylvaticus/Unidentified)	1/-/-			3/2/-			1/1/-			2/4/-			4/3/-		
Calibrated Dating (95,4%)	-------------------			3340-2620 B.C.			-------------------			2930-2700 B.C.			3020-2710 B.C.		

Trench I contexts	Context 156			Context 157			Context 162			Context 164			Context 165			Context 168		
	Obtained MNE	Expected MNE	Relative Abundance	Obtained MNE	Expected MNE	Relative Abundance	Obtained MNE	Expected MNE	Relative Abundance	Obtained MNE	Expected MNE	Relative Abundance	Obtained MNE	Expected MNE	Relative Abundance	Obtained MNE	Expected MNE	Relative Abundance
Maxilla	0	4	0.0%	9	10	90.0%	12	20	60.0%	20	40	50.0%	3	14	21.4%	182	206	88.4%
Mandible	1	4	25.0%	5	10	50.0%	16	20	80.0%	24	40	60.0%	6	14	42.9%	197	206	95.6%
Scapula	0	4	0.0%	0	10	0.0%	1	20	5.0%	1	40	2.5%	0	14	0.0%	4	206	1.9%
Pelvis	0	4	0.0%	1	10	10.0%	6	20	30.0%	11	40	27.5%	4	14	28.6%	43	206	20.9%
Humerus	3	4	75.0%	3	10	30.0%	7	20	35.0%	18	40	45.0%	4	14	28.6%	63	206	30.6%
Ulna	0	4	0.0%	0	10	0.0%	3	20	15.0%	10	40	25.0%	0	14	0.0%	23	206	11.2%
Radius	0	4	0.0%	1	10	10.0%	2	20	10.0%	3	40	7.5%	0	14	0.0%	5	206	2.4%
Femur	1	4	25.0%	3	10	30.0%	6	20	30.0%	20	40	50.0%	2	14	14.3%	61	206	29.6%
Tibia	0	4	0.0%	3	10	30.0%	6	20	30.0%	4	40	10.0%	1	14	7.1%	18	206	8.7%
Vertebra	2	64	3.1%	4	160	2.5%	23	320	7.2%	51	640	8.0%	2	224	0.9%	148	3296	4.5%
Calcaneus	0	4	0.0%	0	10	0.0%	0	20	0.0%	0	40	0.0%	0	14	0.0%	0	206	0.0%
Talus	0	4	0.0%	0	10	0.0%	0	20	0.0%	1	40	2.5%	0	14	0.0%	0	206	0.0%
Ribs	0	52	0.0%	0	130	0.0%	0	260	0.0%	18	520	3.5%	0	182	0.0%	6	2678	0.2%
Metapodials	1	40	2.5%	1	100	1.0%	0	200	0.0%	25	400	6.3%	0	140	0.0%	8	2060	0.4%
Phalanges	0	112	0.0%	0	280	0.0%	0	560	0.0%	2	1120	0.2%	0	392	0.0%	0	5768	0.0%
Isolated Incisors	2	8	25.0%	7	20	35.0%	16	40	40.0%	45	80	56.3%	1	28	3.6%	178	412	43.2%
Isolated Molars	0	24	0.0%	5	60	8.3%	9	120	7.5%	61	240	25.4%	5	84	6.0%	216	1236	17.5%
Average relative frequency (excluding isolated teeth)	8.7%			16.9%			20.2%			19.9%			9.6%			19.6%		
Average relative frequency (all)	9.2%			17.5%			20.6%			22.3%			9.0%			20.9%		
Minimum Number of Individuals (M.arvalis/A.sylvaticus/Unidentified)	2/-/-			4/1/-			6/4/-			15/5/-			5/2/-			102/1/-		
Calibrated Dating (95,4%)	-------------------			-------------------			2920-2690 B.C.			-------------------			2880-2620 B.C.			3350-3020 B.C.		

Overall	Trench II		
	Obtained MNE	Expected MNE	Relative Abundance
Maxilla	67	148	45.3%
Mandible	121	148	81.8%
Scapula	7	148	4.7%
Pelvis	42	148	28.4%
Humerus	60	148	40.5%
Ulna	18	148	12.2%
Radius	15	148	10.1%
Femur	62	148	41.9%
Tibia	32	148	21.6%
Vertebrae	190	2738	6.9%
Calcaneus	0	148	0.0%
Talus	0	148	0.0%
Ribs	6	1924	0.3%
Metapodials	5	1480	0.3%
Phalangies	0	4144	0.0%
Isolated Incisors	131	296	44.3%
Isolated Molars	172	888	19.4%
Minimum Number of Individuals (M.arvalis/A.sylvaticus/Unidentified)	64/10/-		
Calibrated Dating	2920-2460 BC		

Trench II contexts	Context 204			Context 205			Context 209			Context 211		
	Obtained MNE	Expected MNE	Relative Abundance	Obtained MNE	Expected MNE	Relative Abundance	Obtained MNE	Expected MNE	Relative Abundance	Obtained MNE	Expected MNE	Relative Abundance
Maxilla	0	2	0.0%	0	2	0.0%	6	18	33.3%	28	76	36.8%
Mandible	2	2	100.0%	0	2	0.0%	15	18	83.3%	66	76	86.8%
Scapula	0	2	0.0%	0	2	0.0%	0	18	0.0%	2	76	2.6%
Pelvis	0	2	0.0%	0	2	0.0%	2	18	11.1%	15	76	19.7%
Humerus	0	2	0.0%	0	2	0.0%	5	18	2.8%	31	76	40.8%
Ulna	0	2	0.0%	0	2	0.0%	4	18	22.2%	6	76	7.9%
Radius	0	2	0.0%	0	2	0.0%	3	18	16.7%	8	76	10.5%
Femur	1	2	50.0%	2	2	100.0%	3	18	16.7%	29	76	38.2%
Tibia	0	2	0.0%	0	2	0.0%	2	18	11.1%	15	76	19.7%
Vertebra	1	32	3.1%	0	32	0.0%	0	288	0.0%	32	1216	2.6%
Calcaneus	0	2	0.0%	0	2	0.0%	0	18	0.0%	0	76	0.0%
Talus	0	2	0.0%	0	2	0.0%	0	18	0.0%	0	76	0.0%
Ribs	0	26	0.0%	0	26	0.0%	2	234	0.9%	2	988	0.2%
Metapodials	0	20	0.0%	0	20	0.0%	1	180	0.6%	3	760	0.4%
Phalanges	0	56	0.0%	0	56	0.0%	0	504	0.0%	0	2128	0.0%
Isolated Incisors	0	4	0.0%	2	4	50.0%	23	36	63.9%	86	152	56.6%
Isolated Molars	2	12	16.7%	6	12	50.0%	35	108	32.4%	84	456	18.4%
Average relative frequency (excluding isolated teeth)	10.2%			6.7%			13.2%			17.7%		
Average relative frequency (all)	10.0%			11.8%			17.3%			20.0%		
Minimum Number of Individuals (M.arvalis/A.sylvaticus/Unidentified)	1/-/-			1/-/-			9/-/-			36/2/-		
Calibrated Dating	2860-2460 B.C.			-------------------			-------------------			2920-2630 B.C.		

Trench II contexts	Context 213			Context 214			Context 215		
	Obtained MNE	Expected MNE	Relative Abundance	Obtained MNE	Expected MNE	Relative Abundance	Obtained MNE	Expected MNE	Relative Abundance
Maxilla	33	42	78.6%	0	2	0.0%	0	6	0.0%
Mandible	34	42	81.0%	1	2	50.0%	3	6	50.0%
Scapula	5	42	11.9%	0	2	0.0%	0	6	0.0%
Pelvis	25	42	59.5%	0	2	0.0%	0	6	0.0%
Humerus	22	42	52.4%	0	2	0.0%	2	6	33.3%
Ulna	7	42	16.7%	0	2	0.0%	1	6	16.7%
Radius	3	42	7.1%	0	2	0.0%	1	6	16.7%
Femur	25	42	59.5%	1	2	50.0%	1	6	16.7%
Tibia	13	42	31.0%	0	2	0.0%	2	6	33.3%
Vertebra	153	672	22.8%	4	32	12.5%	0	96	0.0%
Calcaneus	0	42	0.0%	0	2	0.0%	0	6	0.0%
Talus	0	42	0.0%	0	2	0.0%	0	6	0.0%
Ribs	1	546	0.2%	0	26	0.0%	1	78	1.3%
Metapodials	0	420	0.0%	0	20	0.0%	1	60	1.7%
Phalanges	0	1176	0.0%	0	56	0.0%	0	168	0.0%
Isolated Incisors	17	84	20.2%	3	4	75.0%	0	12	0.0%
Isolated Molars	40	252	15.9%	0	12	0.0%	5	36	13.9%
Average relative frequency (excluding isolated teeth)	28.0%			7.5%			11.3%		
Average relative frequency (all)	26.9%			11.0%			10.8%		
Minimum Number of Individuals (M.arvalis/A.sylvaticus/Unidentified)	14/7/-			1/-/-			2/1/-		
Calibrated Dating	2920-2670 B.C.			-------------------			2890-2630 B.C.		

Overall	Trench III		
	Obtained MNE	Expected MNE	Relative Abundance
Maxilla	9	50	18.0%
Mandible	18	50	36.0%
Scapula	0	50	0.0%
Pelvis	3	50	6.0%
Humerus	7	50	14.0%
Ulna	3	50	6.0%
Radius	6	50	12.0%
Femur	10	50	20.0%
Tibia	10	50	20.0%
Vertebrae	1	925	0.1%
Calcaneus	0	50	0.0%
Talus	0	50	0.0%
Ribs	2	650	0.3%
Metapodials	4	500	0.8%
Phalangies	0	1400	0.0%
Isolated Incisors	16	100	16.0%
Isolated Molars	10	300	3.3%
Minimum Number of Individuals (M.arvalis/A.sylvaticus/Unidentified)	22/-/3		
Calibrated Dating	<2880BC - >861AD?		

Trench III contexts	Context 325			Context 327			Context 328			Context 333			Context 336			Context 337		
	Obtained MNE	Expected MNE	Relative Abundance	Obtained MNE	Expected MNE	Relative Abundance	Obtained MNE	Expected MNE	Relative Abundance	Obtained MNE	Expected MNE	Relative Abundance	Obtained MNE	Expected MNE	Relative Abundance	Obtained MNE	Expected MNE	Relative Abundance
Maxilla	0	2	0.0%	2	4	50.0%	0	2	0.0%	0	2	0.0%	0	2	0.0%	2	20	10.0%
Mandible	0	2	0.0%	0	4	0.0%	0	2	0.0%	1	2	50.0%	1	2	50.0%	13	20	65.0%
Scapula	0	2	0.0%	0	4	0.0%	0	2	0.0%	0	2	0.0%	0	2	0.0%	0	20	0.0%
Pelvis	0	2	0.0%	1	4	25.0%	0	2	0.0%	0	2	0.0%	0	2	0.0%	0	20	0.0%
Humerus	0	2	0.0%	0	4	0.0%	0	2	0.0%	0	2	0.0%	0	2	0.0%	3	20	15.0%
Ulna	0	2	0.0%	0	4	0.0%	0	2	0.0%	0	2	0.0%	0	2	0.0%	0	20	0.0%
Radius	1	2	50.0%	0	4	0.0%	1	2	50.0%	0	2	0.0%	0	2	0.0%	0	20	0.0%
Femur	0	2	0.0%	3	4	75.0%	1	2	50.0%	0	2	0.0%	0	2	0.0%	4	20	20.0%
Tibia	0	2	0.0%	1	4	25.0%	0	2	0.0%	0	2	0.0%	0	2	0.0%	4	20	20.0%
Vertebra	0	32	0.0%	0	64	0.0%	0	32	0.0%	0	32	0.0%	0	32	0.0%	0	320	0.0%
Calcaneus	0	2	0.0%	0	4	0.0%	0	2	0.0%	0	2	0.0%	0	2	0.0%	0	20	0.0%
Talus	0	2	0.0%	0	4	0.0%	0	2	0.0%	0	2	0.0%	0	2	0.0%	0	20	0.0%
Ribs	0	26	0.0%	0	52	0.0%	0	26	0.0%	0	26	0.0%	0	26	0.0%	0	260	0.0%
Metapodials	0	20	0.0%	0	40	0.0%	0	20	0.0%	0	20	0.0%	0	20	0.0%	0	200	0.0%
Phalanges	0	56	0.0%	0	112	0.0%	0	56	0.0%	0	56	0.0%	0	56	0.0%	0	560	0.0%
Isolated Incisors	0	4	0.0%	0	8	0.0%	0	4	0.0%	0	4	0.0%	0	4	0.0%	14	40	35.0%
Isolated Molars	0	12	0.0%	0	24	0.0%	0	12	0.0%	0	12	0.0%	0	12	0.0%	5	120	4.2%
Average relative frequency (excluding isolated teeth)	3.3%			11.7%			6.7%			3.3%			3.3%			8.7%		
Average relative frequency (all)	2.9%			10.3%			5.9%			2.9%			2.9%			10.0%		
Minimum Number of Individuals (M.arvalis/A.sylvaticus/Unidentified)	-/-/1			2/-/-			1/-/-			1/-/-			-/-/1			10/-/-		
Calibrated Dating	---------------			434 - 861 AD			---------------			---------------			---------------			---------------		

Trench III contexts	Context 338			Context 339			Context 346			Context 364			Context 369		
	Obtained MNE	Expected MNE	Relative Abundance	Obtained MNE	Expected MNE	Relative Abundance	Obtained MNE	Expected MNE	Relative Abundance	Obtained MNE	Expected MNE	Relative Abundance	Obtained MNE	Expected MNE	Relative Abundance
Maxilla	0	2	0.0%	2	4	50.0%	2	2	100.0%	0	6	0.0%	0	4	0.0%
Mandible	0	2	0.0%	0	4	0.0%	0	2	0.0%	4	6	66.7%	0	4	0.0%
Scapula	0	2	0.0%	0	4	0.0%	0	2	0.0%	0	6	0.0%	0	4	0.0%
Pelvis	0	2	0.0%	1	4	25.0%	1	2	50.0%	0	6	0.0%	0	4	0.0%
Humerus	1	2	50.0%	3	4	75.0%	0	2	0.0%	0	6	0.0%	0	4	0.0%
Ulna	0	2	0.0%	2	4	50.0%	0	2	0.0%	0	6	0.0%	1	4	25.0%
Radius	0	2	0.0%	2	4	50.0%	0	2	0.0%	0	6	0.0%	2	4	50.0%
Femur	0	2	0.0%	1	4	25.0%	0	2	0.0%	0	6	0.0%	1	4	25.0%
Tibia	1	2	50.0%	1	4	25.0%	2	2	100.0%	0	6	0.0%	1	4	25.0%
Vertebra	0	32	0.0%	0	64	0.0%	0	32	0.0%	0	96	0.0%	1	64	1.6%
Calcaneus	0	2	0.0%	0	4	0.0%	0	2	0.0%	0	6	0.0%	0	4	0.0%
Talus	0	2	0.0%	0	4	0.0%	0	2	0.0%	0	6	0.0%	0	4	0.0%
Ribs	0	26	0.0%	0	52	0.0%	0	26	0.0%	0	78	0.0%	2	52	3.9%
Metapodials	0	20	0.0%	0	40	0.0%	0	20	0.0%	0	60	0.0%	4	40	10.0%
Phalanges	0	56	0.0%	0	112	0.0%	0	56	0.0%	0	168	0.0%	0	112	0.0%
Isolated Incisors	0	4	0.0%	0	8	0.0%	0	4	0.0%	2	12	16.7%	0	8	0.0%
Isolated Molars	0	12	0.0%	0	24	0.0%	0	12	0.0%	5	36	13.9%	0	24	0.0%
Average relative frequency (excluding isolated teeth)	6.7%			20.0%			16.7%			4.4%			9.4%		
Average relative frequency (all)	5.9%			17.6%			14.7%			5.7%			8.3%		
Minimum Number of Individuals (M.arvalis/A.sylvaticus/Unidentified)	1/-/-			2/-/-			1/-/-			3/-/-			1/-/1		
Calibrated Dating	---------------			---------------			---------------			2300 - 2040 BC			---------------		

Overall	Trench IV		
	Obtained MNE	Expected MNE	Relative Abundance
Maxilla	379	470	80.6%
Mandible	443	470	94.3%
Scapula	150	470	31.9%
Pelvis	271	470	57.7%
Humerus	382	470	81.3%
Ulna	153	470	32.6%
Radius	58	470	12.3%
Femur	370	470	78.7%
Tibia	257	470	54.7%
Vertebrae	1630	8695	18.8%
Calcaneus	20	470	4.3%
Talus	1	470	0.2%
Ribs	12	6110	0.2%
Metapodials	45	4700	1.0%
Phalangies	1	13160	0.008%
Isolated Incisors	528	940	56.2%
Isolated Molars	1039	2820	36.8%
Minimum Number of Individuals (M.arvalis/A.sylvaticus/Unidentified)	224/11		
Calibrated Dating	2860 BC - ?AD		

Trench IV contexts	Context 402			Context 403			Context 405			Context 406		
	Obtained MNE	Expected MNE	Relative Abundance	Obtained MNE	Expected MNE	Relative Abundance	Obtained MNE	Expected MNE	Relative Abundance	Obtained MNE	Expected MNE	Relative Abundance
Maxilla	0	2	0.0%	0	4	0.0%	2	2	100.0%	0	2	0.0%
Mandible	0	2	0.0%	1	4	25.0%	1	2	50.0%	1	2	50.0%
Scapula	0	2	0.0%	0	4	0.0%	0	2	0.0%	0	2	0.0%
Pelvis	0	2	0.0%	2	4	50.0%	0	2	0.0%	0	2	0.0%
Humerus	0	2	0.0%	2	4	50.0%	0	2	0.0%	0	2	0.0%
Ulna	0	2	0.0%	1	4	25.0%	0	2	0.0%	1	2	50.0%
Radius	0	2	0.0%	0	4	0.0%	0	2	0.0%	0	2	0.0%
Femur	1	2	50.0%	0	4	0.0%	0	2	0.0%	0	2	0.0%
Tibia	0	2	0.0%	0	4	0.0%	2	2	100.0%	0	2	0.0%
Vertebra	0	32	0.0%	0	64	0.0%	0	32	0.0%	0	32	0.0%
Calcaneus	0	2	0.0%	0	4	0.0%	0	2	0.0%	0	2	0.0%
Talus	0	2	0.0%	0	4	0.0%	0	2	0.0%	0	2	0.0%
Ribs	0	26	0.0%	0	52	0.0%	0	26	0.0%	0	26	0.0%
Metapodials	0	20	0.0%	0	40	0.0%	0	20	0.0%	0	20	0.0%
Phalanges	0	56	0.0%	0	112	0.0%	0	56	0.0%	0	56	0.0%
Isolated Incisors	0	4	0.0%	0	8	0.0%	2	4	50.0%	1	4	25.0%
Isolated Molars	0	12	0.0%	0	24	0.0%	6	12	50.0%	0	12	0.0%
Average relative frequency (excluding isolated teeth)	3.3%			10.0%			16.7%			6.7%		
Average relative frequency (all)	2.9%			8.8%			20.6%			7.4%		
Minimum Number of Individuals (M.arvalis/A.sylvaticus/Unidentified)	-/1/-			1/1/-			1/-/-			1/-/-		
Calibrated Dating	------------------			------------------			------------------			------------------		

Trench IV contexts	Context 407			Context 408			Context 423			Context 428		
	Obtained MNE	Expected MNE	Relative Abundance	Obtained MNE	Expected MNE	Relative Abundance	Obtained MNE	Expected MNE	Relative Abundance	Obtained MNE	Expected MNE	Relative Abundance
Maxilla	8	66	12.1%	369	384	96.1%	0	6	0.0%	0	4	0.0%
Mandible	60	66	90.9%	370	384	96.4%	6	6	100.0%	4	4	100.0%
Scapula	0	66	0.0%	150	384	39.1%	0	6	0.0%	0	4	0.0%
Pelvis	0	66	0.0%	269	384	70.1%	0	6	0.0%	0	4	0.0%
Humerus	2	66	3.0%	376	384	97.9%	0	6	0.0%	2	4	50.0%
Ulna	1	66	1.5%	150	384	39.1%	0	6	0.0%	0	4	0.0%
Radius	0	66	0.0%	58	384	15.1%	0	6	0.0%	0	4	0.0%
Femur	2	66	3.0%	366	384	95.3%	1	6	16.7%	0	4	0.0%
Tibia	1	66	1.5%	253	384	65.9%	0	6	0.0%	1	4	25.0%
Vertebra	2	1056	0.2%	1628	6144	26.5%	0	96	0.0%	0	64	0.0%
Calcaneus	0	66	0.0%	20	384	5.2%	0	6	0.0%	0	4	0.0%
Talus	0	66	0.0%	1	384	0.3%	0	6	0.0%	0	4	0.0%
Ribs	0	858	0.0%	12	4992	0.2%	0	78	0.0%	0	52	0.0%
Metapodials	0	660	0.0%	45	3840	1.2%	0	60	0.0%	0	40	0.0%
Phalanges	0	1848	0.0%	1	10752	0.01%	0	168	0.0%	0	112	0.0%
Isolated Incisors	40	132	30.3%	479	768	62.4%	4	12	33.3%	2	8	25.0%
Isolated Molars	74	396	18.7%	955	2304	41.5%	4	36	11.1%	0	24	0.0%
Average relative frequency (excluding isolated teeth)	7.5%			43.2%			7.8%			11.7%		
Average relative frequency (all)	9.5%			44.2%			9.5%			11.8%		
Minimum Number of Individuals (M.arvalis/A.sylvaticus/Unidentified)	33/-/-			183/9/-			3/-/-			2/-/-		
Calibrated Dating	2460 - 2200 BC			------------------			------------------			------------------		
